# Physical activity and sleep quality among college students: a cross-sectional analysis of bedtime procrastination, social jetlag, and chronotype

**DOI:** 10.3389/fpubh.2026.1949891

**Published:** 2026-09-15

**Authors:** Jiameng Sun, Rui Li, Jie Wu, Qiqi Zhou, Mohamad Nizam Nazarudin

**Affiliations:** 1 Institute of Physical Education, Nantong Normal College, Nantong, China; 2 Faculty of Education, Universiti Kebangsaan Malaysia, Bangi, Selangor, Malaysia; 3 Institute of Sports Science, Nantong University, Nantong, China; 4 Nantong Health Vocational College, Nantong, China

**Keywords:** bedtime procrastination, chronotype, college students, physical activity, public health, sleep quality, social jetlag

## Abstract

**Background:**

Sleep problems are common among college students and may involve daytime behavior, bedtime self-regulation, and irregular sleep timing. This study examined the associations of physical activity, bedtime procrastination, social jetlag, and chronotype with sleep quality, including indirect associations through bedtime procrastination and social jetlag and whether these varied by chronotype.

**Methods:**

This cross-sectional study included 1,058 college students from four provinces in eastern China. Physical activity, sleep quality, bedtime procrastination, social jetlag, and chronotype were assessed using the Physical Activity Rating Scale-3, Pittsburgh Sleep Quality Index (PSQI), Bedtime Procrastination Scale, selected sleep-timing items from the Munich Chronotype Questionnaire, and five-item Morningness–Eveningness Questionnaire, respectively. Correlation and regression analyses examined direct, indirect, and conditional associations.

**Results:**

Physical activity was negatively correlated with PSQI scores (*r* = –0.383, *p* < 0.001), bedtime procrastination (*r* = –0.505, *p* < .001), and social jetlag (*r* = –0.420, *p* < 0.001). After adjustment for covariates and other model variables, physical activity remained negatively associated with PSQI scores (B = −0.014, *p* = 0.003). The indirect association through bedtime procrastination was negative [B = −0.017, 95% bootstrap CI (−0.023, −0.011)]. The indirect association through social jetlag was negative at more evening-oriented and mean chronotype scores (B = −0.017 and −0.005, respectively) but positive at a more morning-oriented score (B = 0.007). The serial indirect association through bedtime procrastination and social jetlag showed the same pattern (B = −0.023, −0.007, and 0.009, respectively).

**Conclusion:**

Higher physical activity was directly associated with lower PSQI scores and indirectly associated with PSQI scores through less bedtime procrastination. Indirect associations involving social jetlag depended on chronotype, being negative at more evening-oriented and mean chronotype scores but reversing at a more morning-oriented score. These findings highlight the joint relevance of physical activity, bedtime regulation, sleep-timing regularity, and chronotype to sleep quality. Because the study was cross-sectional, the observed associations should not be interpreted as temporal or causal effects.

## 1 Introduction

Sleep problems are common among college students and are closely related to mental health, daytime functioning, and academic adjustment (1, 2). The transition to college brings greater control over daily schedules. It also brings irregular routines, academic stress, dormitory disturbances, and frequent electronic media use at night. Screen use at bedtime has been associated with poorer sleep outcomes (3). Bedtime media use and sleep problems may also reinforce each other over time (4, 5). Insufficient sleep and daytime sleepiness may further affect learning, memory, and general health (6). Sleep problems in this population therefore involve more than sleep duration. Daytime behavior, bedtime regulation, and sleep-timing regularity may also be relevant.

Physical activity is 1 daytime behavior that has been consistently associated with sleep. Reviews and meta-analyses have reported associations between physical activity or exercise and several sleep outcomes, although the magnitude of these associations may vary by activity type, intensity, timing, population, and study design (7–13). Similar findings have been reported specifically among university students (14). Research in Chinese college students has also linked physical activity and sleep quality through factors such as smartphone use and self-control (15, 16). The two-process model of sleep provides one explanation for these associations. Sleep–wake regulation involves both homeostatic sleep pressure and circadian timing (17). Physical activity may form part of a student's daily routine and daytime rhythm. More active students may follow more regular schedules, whereas lower activity may occur alongside prolonged sedentary time and later evening wakefulness. A direct association alone, however, does not show which bedtime behaviors or sleep-timing patterns are involved.

Bedtime procrastination may be one relevant behavioral factor. It refers to going to bed later than intended despite the absence of external barriers (18). It is usually viewed as a problem of self-regulation rather than simply a preference for a late bedtime (19). College students have considerable control over their evening time. Smartphone use, gaming, videos, and delayed academic work may interfere with sleep preparation. Difficulty stopping these activities can lead to later bedtimes (20). Bedtime procrastination has been associated with poorer sleep quality among Chinese college students (21). Similar findings have been reported in studies of mobile phone dependence, fear of missing out, and sleep hygiene (22, 23). Physical activity may also be related to this form of regulation. Students with more structured daytime routines may find it easier to stop evening activities and prepare for sleep. Eveningness, lower self-control, and procrastination have also been linked in college students (24, 25).

Social jetlag may provide a rhythm-related explanation. It refers to the difference in sleep midpoint between school days and free days (26). College schedules often require early wake times on school days, whereas free days allow more flexible sleep timing. This may produce later bedtimes, later wake times, and catch-up sleep. Daily activities and social schedules also act as time cues for the circadian system (27). Exercise may serve as a non-photic circadian cue, although its effects depend on exercise timing and individual circadian state (28). Students with more regular activity schedules may therefore show smaller differences between school-day and free-day sleep timing. In contrast, low activity may occur with more sedentary time and later routines.

Bedtime procrastination and social jetlag describe different aspects of sleep behavior, but they may be connected. Bedtime procrastination concerns delayed sleep preparation on a given night. Social jetlag concerns differences in sleep timing across the week. Repeated bedtime delay may widen the gap between school days and free days, especially when students sleep later on free days but must wake early for classes. Higher physical activity may therefore be associated with less bedtime procrastination, which may be associated with smaller social jetlag and lower PSQI scores. This proposed sequence links daytime activity, bedtime behavior, weekly sleep timing, and sleep quality.

Chronotype may change the association between social jetlag and sleep quality. Morning-type individuals usually prefer earlier sleep and wake times, whereas evening-type individuals prefer later schedules. Early classes and fixed university schedules may be more difficult for evening-type students to follow. These students may wake earlier than preferred on school days and return to later schedules on free days. The same degree of social jetlag may therefore have different implications across chronotypes. Previous studies have linked social jetlag and chronotype with depressive symptoms, sleep quality, and academic burnout among college students (29–31). The association between social jetlag and PSQI scores may be stronger among students with a greater eveningness tendency.

Several questions remain. Few studies have examined bedtime procrastination and social jetlag within the same model. Their possible sequential association also remains unclear. In addition, limited evidence is available on whether chronotype changes the association between social jetlag and sleep quality or the indirect associations involving social jetlag. The present study examined these questions among college students. It tested the associations among physical activity, bedtime procrastination, social jetlag, chronotype, and PSQI scores. It also examined serial indirect associations through bedtime procrastination and social jetlag. Finally, it tested the moderating role of chronotype. Because all variables were measured at the same time point, the proposed paths represent theoretical and statistical associations rather than temporal or causal relationships.

The following hypotheses were tested:

*H1: Higher physical activity is associated with lower PSQI scores*.
*H2: Physical activity is indirectly associated with PSQI scores through bedtime procrastination. Higher physical activity is associated with less bedtime procrastination, which is associated with lower PSQI scores*.
*H3a: Physical activity is indirectly associated with PSQI scores through social jetlag. Higher physical activity is associated with smaller social jetlag, which is associated with lower PSQI scores*.
*H3b: Physical activity is serially indirectly associated with PSQI scores through bedtime procrastination and social jetlag. Higher physical activity is associated with less bedtime procrastination. Less bedtime procrastination is associated with smaller social jetlag, which is associated with lower PSQI scores*.
*H4: Chronotype moderates the association between social jetlag and PSQI scores. The positive association is expected to be stronger among students with a greater eveningness tendency*.
*H5: Chronotype moderates the indirect associations involving social jetlag. These associations are expected to be stronger among students with a greater eveningness tendency*.

## 2 Materials and methods

### 2.1 Study design and participants

This cross-sectional online survey recruited university students from universities in four provinces in eastern China. Both undergraduate and postgraduate students were eligible to participate. Convenience sampling was used, and all study variables were assessed at one time point. The design therefore allowed the examination of associations, but not temporal order or causality. Participants were eligible if they were 17–24 years old, currently enrolled at a university, able to complete the questionnaire independently, and willing to participate. Responses were screened according to the following exclusion criteria: unusually short completion time, repetitive or invariant response patterns, missing data on the primary study variables, implausible age or sleep-timing values, and insufficient information to calculate social jetlag or the main scale scores. A total of 1,100 questionnaires were returned. After data screening, 42 responses were excluded. The final analytic sample comprised 1,058 participants, corresponding to an analytic retention rate of 96.2%. Of these participants, 592 were male (56.0%) and 466 were female (44.0%). Their ages ranged from 17 to 24 years, with a mean age of 20.07 years (SD = 1.55). Sex, age, caffeine intake frequency, smoking or e-cigarette use frequency, alcohol use frequency, academic stress, and dormitory environmental disturbance were included as covariates.

### 2.2 Measures

#### 2.2.1 Physical activity

Physical activity was assessed using the Physical Activity Rating Scale-3 [PARS-3, (32)]. The scale assesses exercise intensity, duration, and frequency. A total score was calculated as follows: intensity × (duration – 1) × frequency. Higher scores indicate higher levels of physical activity. The PARS-3 has been widely used among Chinese college students (33). The total score was used in all analyses.

#### 2.2.2 Sleep quality

Sleep quality was assessed using the Pittsburgh Sleep Quality Index [PSQI, (34)]. The PSQI assesses sleep during the previous month. It includes seven components: subjective sleep quality, sleep latency, sleep duration, sleep efficiency, sleep disturbances, use of sleep medication, and daytime dysfunction. The Chinese version has shown acceptable reliability and validity (35). Higher total scores indicate poorer sleep quality. In the present sample, Cronbach's α was 0.914. The total PSQI score was used as the outcome variable.

#### 2.2.3 Bedtime procrastination

Bedtime procrastination was assessed using the nine-item Bedtime Procrastination Scale (BPS, 18). Each item is rated from 1 (“never”) to 5 (“always”). Reverse-coded items were recoded before the item scores were averaged. Higher scores indicate greater bedtime procrastination. The Chinese version has shown acceptable reliability and validity among college students (36). In the present sample, Cronbach's α was 0.910.

#### 2.2.4 Social jetlag

Social jetlag was calculated using sleep-timing items adapted from the Munich Chronotype Questionnaire [MCTQ, (37)]. The MCTQ has been validated among Chinese college students (38). The original workday and free-day categories were adapted to school days and free days to reflect university schedules. Participants reported their usual sleep timing on school days and free days during the previous month. Sleep onset was calculated by adding sleep latency to the reported time at which the participant began trying to sleep. Sleep midpoints were then calculated separately for school days and free days as the midpoint between sleep onset and wake time. To account for midnight crossing, social jetlag was calculated as the smaller circular difference between the two sleep midpoints: SJL = min (|MSF – MSW|, 24 – |MSF – MSW|), where MSF is the sleep midpoint on free days and MSW is the sleep midpoint on school days. Social jetlag was expressed in hours. Higher values indicate a larger difference in sleep timing between school days and free days.

#### 2.2.5 Chronotype

Chronotype was assessed using the five-item Morningness–Eveningness Questionnaire [MEQ-5, (39)]. The MEQ-5 was selected because it provides a brief assessment of morningness–eveningness and reduces respondent burden in a multi-measure online survey. This was particularly relevant because participants were required to complete several behavioral and sleep-related measures in the same survey. The scale assesses preferred wake time, alertness after waking, evening sleepiness, preferred time for peak performance, and self-rated morningness or eveningness. Total scores range from 4 to 25. Higher scores indicate greater morningness, whereas lower scores indicate greater eveningness. The Chinese version has shown acceptable reliability and validity in student samples (40). In the present sample, Cronbach's α was 0.755. The continuous total score was used in all primary analyses. For descriptive purposes only, participants were also classified according to the original reduced MEQ scoring criteria as evening types (scores 4–11), neither types (scores 12–17), or morning types (scores 18–25).

#### 2.2.6 Covariates

The covariates were sex, age, caffeine intake frequency, smoking or e-cigarette use frequency, alcohol use frequency, academic stress, and dormitory environmental disturbance. These variables were included because demographic characteristics, stimulant and substance use, academic stress, and the dormitory environment may be associated with sleep quality or sleep timing among university students and could therefore confound the associations of interest (41–43). Sex was coded as 1 = male and 2 = female. Age was entered in years. Caffeine intake frequency was rated from 1 (“almost never”) to 5 (“twice per day or more”). Smoking or e-cigarette use frequency was rated from 1 (“none”) to 4 (“almost every day”). Alcohol use frequency was rated from 1 (“never”) to 5 (“three times per week or more”). Academic stress and dormitory environmental disturbance were each rated from 1 to 5, with higher scores indicating greater stress or disturbance.

### 2.3 Procedure and ethics

Data were collected through an online questionnaire platform. The survey included demographic information and measures of physical activity, sleep quality, bedtime procrastination, social jetlag, and chronotype. Before data collection, the research team reviewed the questionnaire wording, item order, response options, and response logic. The first survey page explained the study purpose and procedures. It also stated that participation was voluntary and that participants could withdraw at any time. Participants aged 18 years or older provided electronic informed consent before completing the questionnaire. Participants younger than 18 years provided electronic assent, and electronic informed consent was obtained from a parent or legal guardian. No names, student identification numbers, telephone numbers, or other direct identifiers were collected. The study was reviewed and approved by the Ethics Committee of Nantong University (approval number: TD-2024-109).

### 2.4 Statistical analysis

Analyses were conducted using IBM SPSS Statistics version 27.0. The moderated serial indirect association model was examined using PROCESS macro version 4.2, Model 87. PROCESS estimates conditional process models through a series of ordinary least squares regression equations; therefore, the present analysis was regression-based rather than structural equation modeling. Means, standard deviations, skewness, and kurtosis were calculated for the continuous variables. Distributional characteristics were assessed using skewness and kurtosis rather than formal normality tests. Absolute skewness values below 1 and absolute kurtosis values below 2 were considered to indicate no substantial departure from normality. Frequencies and percentages were calculated for categorical variables. Pearson correlations were used to examine bivariate associations among physical activity, bedtime procrastination, social jetlag, chronotype, and PSQI scores. Four regression equations were estimated within the conditional process model. Model 1 examined the association between physical activity and bedtime procrastination. Model 2 examined physical activity and bedtime procrastination as predictors of social jetlag. Model 3 estimated the adjusted total association between physical activity and PSQI scores. Model 4 included physical activity, bedtime procrastination, social jetlag, chronotype, and the social jetlag × chronotype interaction as predictors of PSQI scores. These equations represented components of the prespecified conditional process model rather than competing candidate models. Therefore, model selection criteria such as the Akaike information criterion (AIC) were not used. All models adjusted for sex, age, caffeine intake frequency, smoking or e-cigarette use frequency, alcohol use frequency, academic stress, and dormitory environmental disturbance. Social jetlag and chronotype were mean-centered before the interaction term was calculated. Variance inflation factors and tolerance values were examined to assess multicollinearity. Chronotype was retained as a continuous moderator. To probe significant conditional associations, estimates were calculated at one standard deviation below the mean, at the mean, and one standard deviation above the mean of the MEQ-5 score. These values were used as representative points along the continuous chronotype distribution and did not represent categorical chronotype groups. Conditional indirect associations were tested using 5,000 bootstrap samples. An indirect association was considered statistically significant when its 95% bootstrap confidence interval did not include zero. Simple slope analyses were used to interpret significant interactions. All statistical tests were two-sided, with *p* < .05 indicating statistical significance.

## 3 Results

### 3.1 Common method bias assessment

Because the main variables were assessed using self-report measures, Harman's single-factor test was conducted as a preliminary assessment of common method bias. An unrotated principal component analysis identified four factors with eigenvalues greater than 1. The first factor accounted for 43.948% of the total variance. This value was below the commonly used 50% threshold (44). The result did not indicate that a single factor dominated the covariance among the measures. However, Harman's test cannot rule out common method bias.

### 3.2 Descriptive statistics and bivariate correlations

Participant characteristics, descriptive statistics, and comparisons between male and female students are presented in Table 1. The mean physical activity score was 40.67 (SD = 30.50), and the mean PSQI score was 7.91 (SD = 5.43). The mean social jetlag was 2.24 h (SD = 0.46). The mean chronotype score was 13.07 (SD = 4.95). Based on the descriptive MEQ-5 classification, 409 participants (38.7%) were classified as evening types, 429 (40.5%) as neither types, and 220 (20.8%) as morning types. The mean bedtime procrastination score was 2.49 (SD = 0.94). Based on the skewness and kurtosis criteria specified in the statistical analysis, the continuous variables showed no substantial departures from normality; all absolute skewness values were below 1 and all absolute kurtosis values were below 2.

**Table 1:** Participant characteristics and descriptive statistics.

Characteristic or variable	Total (*N* = 1,058)	Male (*n* = 592)	Female (*n* = 466)	*t*/χ^2^	*p*
Year of study				20.850	<0.001
First year	187 (17.7)	105 (17.7)	82 (17.6)	—	—
Second year	261 (24.7)	143 (24.2)	118 (25.3)	—	—
Third year	271 (25.6)	166 (28.0)	105 (22.5)	—	—
Fourth year	245 (23.2)	145 (24.5)	100 (21.5)	—	—
Postgraduate	94 (8.9)	33 (5.6)	61 (13.1)	—	—
Age, years	20.07 (1.55)	19.96 (1.43)	20.19 (1.68)	−2.349	0.019
Caffeine intake frequency	2.32 (1.11)	2.24 (1.09)	2.42 (1.14)	−2.513	0.012
Smoking or e-cigarette use frequency	2.00 (0.99)	2.01 (0.99)	1.98 (1.01)	0.442	0.659
Alcohol use frequency	2.32 (1.26)	2.30 (1.22)	2.35 (1.32)	−0.572	0.567
Academic stress	3.32 (1.21)	3.25 (1.20)	3.40 (1.20)	−1.979	0.048
Dormitory environmental disturbance	2.73 (1.19)	2.66 (1.21)	2.82 (1.16)	−2.216	0.027
Physical activity	40.67 (30.50)	46.63 (30.97)	33.10 (28.17)	7.419	<0.001
PSQI score	7.91 (5.43)	6.76 (5.29)	9.36 (5.26)	−7.973	<0.001
Social jetlag, hours	2.24 (0.46)	2.20 (0.46)	2.29 (0.46)	−3.285	0.001
Chronotype	13.07 (4.95)	12.84 (4.94)	13.35 (4.94)	−1.662	0.097
Chronotype category				4.434	0.109
Evening type (E-type)	409 (38.7)	240 (40.5)	169 (36.3)		
Neither type (N-type)	429 (40.5)	242 (40.9)	187 (40.1)		
Morning type (M-type)	220 (20.8)	110 (18.6)	110 (23.6)		
Bedtime procrastination mean score	2.49 (0.94)	2.36 (0.88)	2.66 (0.98)	−5.271	<0.001

Values are presented as n (%) for categorical variables and M (SD) for continuous or ordinal variables. Percentages within the male and female columns are column percentages. Differences between male and female students were examined using chi-square tests for year of study and chronotype category and Welch's independent-samples t tests for continuous and ordinal variables. Higher PSQI scores indicate poorer sleep quality. Higher MEQ-5 scores indicate greater morningness. For descriptive classification, MEQ-5 scores of 4–11 were classified as evening type, scores of 12–17 as neither type, and scores of 18–25 as morning type. Chronotype categories were used for descriptive presentation only; the continuous MEQ-5 score was retained in all primary analyses.

Zero-order bivariate correlations among the primary study variables are presented in Table 2. Physical activity was negatively correlated with PSQI scores (*r* = – 0.383, *p* < 0.001), social jetlag (*r* = – 0.420, *p* < 0.001), and bedtime procrastination (*r* = – 0.505, *p* < 0.001). Bedtime procrastination was positively correlated with social jetlag (*r* = 0.575, *p* < 0.001) and PSQI scores (*r* =.464, *p* < 0.001). Social jetlag was also positively correlated with PSQI scores (*r* = 0.370, *p* < 0.001).

**Table 2:** Zero-order bivariate correlations among the primary study variables.

Variable	1	2	3	4	5
1. Physical activity	1				
2. PSQI score	−0.383^**^	1			
3. Bedtime procrastination	−0.505^**^	0.464^**^	1		
4. Social jetlag	−0.420^**^	0.370^**^	0.575^**^	1	
5. Chronotype	0.019	0.096^**^	−0.070^*^	−0.054	1

*N* = 1,058. Pearson correlation coefficients are reported. Higher PSQI scores indicate poorer sleep quality. Higher bedtime procrastination scores indicate greater bedtime procrastination. Higher social jetlag values indicate larger differences in sleep midpoint between school days and free days. Higher MEQ-5 scores indicate greater morningness. ^*^p < 0.05; ^**^p < 0.01.

Chronotype was weakly positively correlated with PSQI scores (*r* = 0.096, *p* = 0.002) and weakly negatively correlated with bedtime procrastination (*r* = –.070, *p* = 0.022). Chronotype was not significantly correlated with physical activity (*r* = 0.019, *p* = 0.541) or social jetlag (*r* = –.054, *p* = 0.079).

### 3.3 Regression results for the moderated serial indirect association model

The regression results are presented in Table 3, and the estimated paths of the conditional process model are summarized in Figure 1. All models adjusted for sex, age, caffeine intake frequency, smoking or e-cigarette use frequency, alcohol use frequency, academic stress, and dormitory environmental disturbance. Variance inflation factors ranged from 1.00 to 1.79, and tolerance values ranged from 0.56 to 1.00. These values did not indicate serious multicollinearity.

**Table 3:** Regression results for the moderated serial indirect association model.

Predictor	Model 1: Bedtime procrastination	Model 2: Social jetlag	Model 3: PSQI total association	Model 4: PSQI full model
Sex	0.085 (0.051)	−0.027 (0.023)	1.456 (0.298)^***^	1.107 (0.246)^***^
Age	0.043 (0.016)^**^	0.038 (0.007)^***^	0.425 (0.094)^***^	0.246 (0.079)^**^
Caffeine intake frequency	0.036 (0.022)	0.021 (0.010)^*^	0.718 (0.130)^***^	0.540 (0.107)^***^
Smoking or e-cigarette use frequency	0.025 (0.025)	0.018 (0.011)	0.358 (0.145)^*^	0.191 (0.120)
Alcohol use frequency	0.010 (0.020)	0.015 (0.009)	0.177 (0.114)	0.100 (0.094)
Academic stress	−0.006 (0.021)	0.010 (0.009)	0.632 (0.120)^***^	0.551 (0.099)^***^
Dormitory environmental disturbance	0.051 (0.021)^*^	0.018 (0.010)	0.673 (0.122)^***^	0.321 (0.102)^**^
Physical activity	−0.0147 (0.0008)^***^	−0.0025 (0.0004)^***^	−0.0536 (0.0049)^***^	−0.0138 (0.0047)^**^
Bedtime procrastination	—	0.228 (0.014)^***^	—	1.151 (0.168)^***^
Social jetlag	—	—	—	2.071 (0.333)^***^
Chronotype	—	—	—	0.135 (0.024)^***^
Social jetlag × chronotype	—	—	—	−0.960 (0.054)^***^
*R^2^*	0.271	0.380	0.266	0.506
Adjusted *R^2^*	0.266	0.375	0.260	0.500
*F*	48.791^***^	71.412^***^	47.521^***^	89.046^***^
df	8, 1,049	9, 1,048	8, 1,049	12, 1,045

Values are unstandardized regression coefficients, with standard errors in parentheses. Model 1 predicted bedtime procrastination. Model 2 predicted social jetlag. Model 3 estimated the adjusted total association between physical activity and PSQI scores. Model 4 included bedtime procrastination, social jetlag, chronotype, and the social jetlag × chronotype interaction. All models adjusted for sex, age, caffeine intake frequency, smoking or e-cigarette use frequency, alcohol use frequency, academic stress, and dormitory environmental disturbance. Social jetlag and chronotype were mean-centered before the interaction was tested. Higher PSQI scores indicate poorer sleep quality. ^*^p < 0.05; ^**^ *p* < 0.01; ^***^p < 0.001.

**Figure 1:**
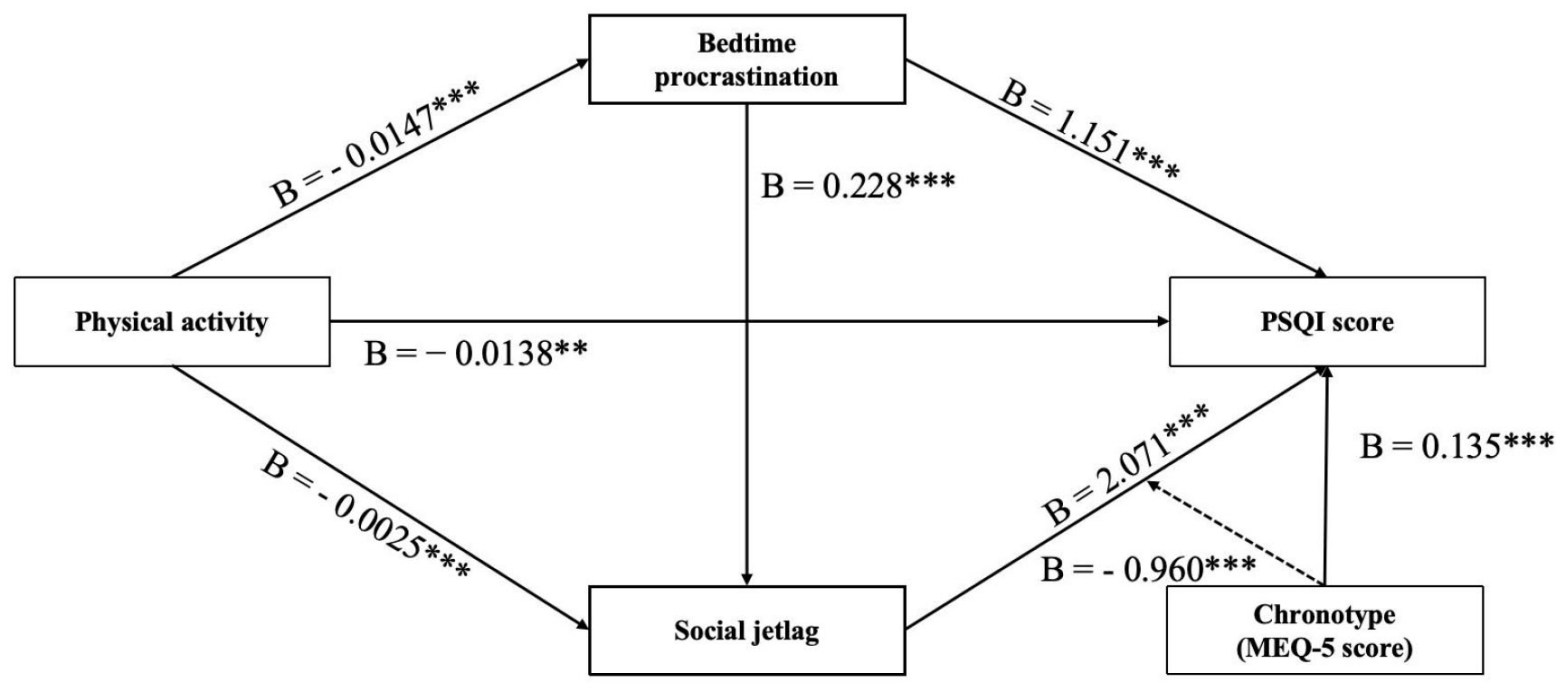
Regression-based conditional process model linking physical activity, bedtime procrastination, social jetlag, chronotype, and PSQI scores. Values shown are unstandardized regression coefficients from the full conditional process model, except for paths predicting bedtime procrastination and social jetlag, which correspond to their respective regression equations. Chronotype moderated the association between social jetlag and PSQI scores. The coefficient for social jetlag represents its conditional association with PSQI scores at the mean chronotype score. All regression equations were adjusted for sex, age, caffeine intake frequency, smoking or e-cigarette use frequency, alcohol use frequency, academic stress, and dormitory environmental disturbance. Covariates are not shown for clarity. *p < .05; **p < .01; ***p < .001. The adjusted total association between physical activity and PSQI scores estimated in Model 3 was B = −0.0536, *p* < .001; the path shown from physical activity to PSQI scores in the figure represents the direct association from the full model (B = −0.0138, *p* =.003).

In Model 1, physical activity was negatively associated with bedtime procrastination (B = −0.0147, SE = 0.0008, *t* = −17.416, *p* < 0.001). The model explained 27.1% of the variance in bedtime procrastination (*R*^2^ = 0.271, adjusted *R*^2^ = 0.266).

In Model 2, physical activity was negatively associated with social jetlag (B = −0.0025, SE = 0.0004, *t* = −5.674, *p* < 0.001). Bedtime procrastination was positively associated with social jetlag (B = 0.228, SE = 0.014, *t* = 16.317, *p* < 0.001). The model explained 38.0% of the variance in social jetlag (*R*^2^ = 0.380, adjusted *R*^2^ = 0.375).

Model 3 estimated the adjusted total association between physical activity and PSQI scores. Physical activity was negatively associated with PSQI scores (B = −0.0536, SE = 0.0049, *t* = −10.957, *p* < 0.001). The model explained 26.6% of the variance in PSQI scores (*R*^2^ = 0.266, adjusted *R*^2^ = 0.260).

Model 4 included bedtime procrastination, social jetlag, chronotype, and the social jetlag × chronotype interaction. Physical activity remained negatively associated with PSQI scores (B = −0.0138, SE = 0.0047, *t* = −2.953, *p* = 0.003). Bedtime procrastination was positively associated with PSQI scores (B = 1.151, SE = 0.168, *t* = 6.835, *p* < 0.001). At the mean chronotype score, social jetlag was positively associated with PSQI scores (B = 2.071, SE = 0.333, *t* = 6.215, *p* < 0.001). At the mean level of social jetlag, chronotype was positively associated with PSQI scores (B = 0.135, SE = 0.024, *t* = 5.590, *p* < 0.001). The social jetlag × chronotype interaction was negative and statistically significant (B = −0.960, SE = 0.054, *t* = −17.846, *p* < 0.001).

Model 4 explained 50.6% of the variance in PSQI scores (*R*^2^ = 0.506, adjusted *R*^2^ = 0.500). Compared with Model 3, the additional variables increased the explained variance by 24.0% (Δ*R*^2^ = 0.240).

### 3.4 Direct, indirect, and moderated indirect associations

The bootstrap results are presented in Table 4. After the mediators, chronotype, and the interaction term were included, physical activity remained negatively associated with PSQI scores [B = −0.014, SE = 0.005, *p* = 0.003, 95% CI (−0.023, −0.005)]. The indirect association through bedtime procrastination was negative and statistically significant [B = −0.017, BootSE = 0.003, 95% bootstrap CI (−0.023, −0.011)]. The indirect association through social jetlag varied by chronotype. At a more evening-oriented chronotype score (M – 1 SD; MEQ-5 = 8.12), the estimate was −0.017 [BootSE = 0.003, 95% bootstrap CI (−0.024, −0.011)]. At the mean chronotype score (MEQ-5 = 13.07), the estimate was −0.005 [BootSE = 0.001, 95% bootstrap CI (−0.008, −0.003)]. At a more morning-oriented chronotype score (M + 1 SD; MEQ-5 = 18.01), the estimate was 0.007 [BootSE = 0.001, 95% bootstrap CI (0.004, 0.010)]. The serial indirect association through bedtime procrastination and social jetlag showed the same change in direction. The estimate was −0.023 at the more evening-oriented chronotype score [BootSE = 0.002, 95% bootstrap CI (−0.028, −0.018)], −0.007 at the mean chronotype score [BootSE = 0.001, 95% bootstrap CI (−0.009, −0.005)], and 0.009 at the more morning-oriented chronotype score [BootSE = 0.002, 95% bootstrap CI (0.006, 0.012)]. The index of moderated indirect association through social jetlag was 0.002 [95% bootstrap CI (0.002, 0.003)]. The index for the serial pathway through bedtime procrastination and social jetlag was 0.003 [95% bootstrap CI (0.003, 0.004)]. Both confidence intervals excluded zero, indicating that the indirect associations involving social jetlag varied across chronotype scores.

**Table 4:** Bootstrap estimates of direct, indirect, and moderated indirect associations.

Association type	Pathway	Chronotype condition	Estimate	SE/BootSE	95% CI
Direct association	Physical activity → PSQI score	—	−0.014	0.005	[−0.023, −0.005]
Indirect association	Physical activity → Bedtime procrastination → PSQI score	—	−0.017	0.003	[−0.023, −0.011]
Conditional indirect association	Physical activity → Social jetlag → PSQI score	More evening-oriented chronotype score: *M* – 1 SD (MEQ-5 = 8.12)	−0.017	0.003	[−0.024, −0.011]
		Mean chronotype score (MEQ-5 = 13.07)	−0.005	0.001	[−0.008, −0.003]
		More morning-oriented chronotype score: *M* + 1 SD (MEQ-5 = 18.01)	0.007	0.001	[0.004, 0.010]
Conditional serial indirect association	Physical activity → Bedtime procrastination → Social jetlag → PSQI score	More evening-oriented chronotype score: *M* – 1 SD (MEQ-5 = 8.12)	−0.023	0.002	[−0.028, −0.018]
		Mean chronotype score (MEQ-5 = 13.07)	−0.007	0.001	[−0.009, −0.005]
		More morning-oriented chronotype score: *M* + 1 SD (MEQ-5 = 18.01)	0.009	0.002	[0.006, 0.012]
Index of moderated indirect association	Physical activity → Social jetlag → PSQI score	—	0.002	<0.001	[0.002, 0.003]
Index of moderated serial indirect association	Physical activity → Bedtime procrastination → Social jetlag → PSQI score	—	0.003	<0.001	[0.003, 0.004]

Unstandardized estimates are reported. The direct-association row reports the conventional standard error; all indirect-association rows report bootstrap standard errors. Conditional estimates were calculated at the mean of the continuous MEQ-5 score and at one standard deviation below and above the mean. For clarity, these probe values are described as more evening-oriented, mean, and more morning-oriented chronotype scores, respectively; they do not represent discrete chronotype groups. Lower MEQ-5 scores indicate greater eveningness, whereas higher MEQ-5 scores indicate greater morningness. Bootstrap confidence intervals were based on 5,000 resamples. An indirect association was considered statistically significant when its 95% bootstrap confidence interval did not include zero.

### 3.5 Conditional association between social jetlag and PSQI scores

The significant social jetlag × chronotype interaction was examined using conditional effect analyses. The results are presented in Table 5 and Figure 2. At a more evening-oriented chronotype score (M – 1 SD; MEQ-5 = 8.12), social jetlag was positively associated with PSQI scores [B = 6.819, SE = 0.467, *t* = 14.597, *p* < 0.001, 95% CI (5.902, 7.736)]. At the mean chronotype score (M; MEQ-5 = 13.07), the association remained positive but was smaller [B = 2.071, SE = 0.333, *t* = 6.215, *p* < .001, 95% CI (1.417, 2.725)]. At a more morning-oriented chronotype score (M + 1 SD; MEQ-5 = 18.01), the direction of the association was reversed. Social jetlag was negatively associated with PSQI scores [B = −2.676, SE = 0.381, *t* = −7.017, *p* < .001, 95% CI (−3.425, −1.928)]. For visualization, the predicted PSQI scores were calculated at one standard deviation below and above the mean of social jetlag. At the more evening-oriented chronotype score, the predicted PSQI score increased from 3.987 at lower social jetlag to 10.261 at higher social jetlag. At the more morning-oriented chronotype score, the predicted score decreased from 9.688 to 7.226. The interaction therefore involved both a change in the strength and a reversal in the direction of the association. Taken together, H1 and H2 were supported. H3a and H3b received conditional support because the expected negative indirect associations were observed at more evening-oriented and mean chronotype scores but reversed at a more morning-oriented chronotype score. H4 and H5 were partially supported because the expected risk pattern was observed at greater eveningness, whereas the direction was reversed at greater morningness.

**Table 5:** Conditional associations between social jetlag and PSQI scores at selected chronotype values.

Chronotype condition	Centered MEQ-5 value	Raw MEQ-5 score	B	SE	*t*	*p*	95% CI
More evening-oriented chronotype score (M – 1 SD)	−4.946	8.12	6.819	0.467	14.597	<.001	[5.902, 7.736]
Mean chronotype score (M)	0	13.07	2.071	0.333	6.215	<.001	[1.417, 2.725]
More morning-oriented chronotype score (M + 1 SD)	4.946	18.01	−2.676	0.381	−7.017	<.001	[−3.425, −1.928]

Values represent the unstandardized conditional association between social jetlag and PSQI scores at selected values of the continuous MEQ-5 score. The M – 1 SD, mean, and M + 1 SD values are described as more evening-oriented, mean, and more morning-oriented chronotype scores, respectively, and do not represent discrete chronotype groups. Chronotype and social jetlag were mean-centered before the interaction was calculated. Higher PSQI scores indicate poorer sleep quality.

**Figure 2:**
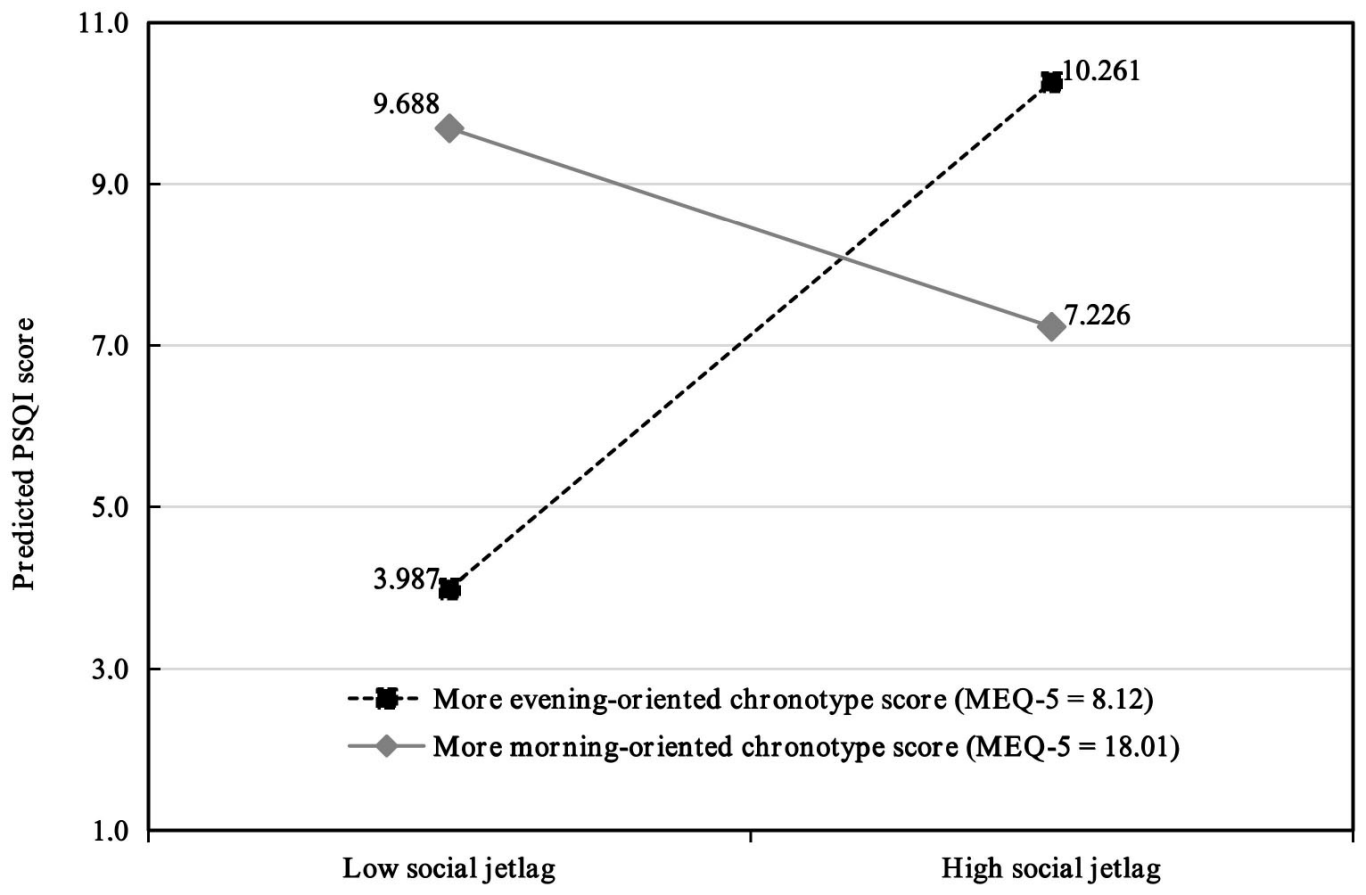
Conditional association between social jetlag and predicted PSQI scores at more evening-oriented and more morning-oriented chronotype scores. Predicted PSQI scores were calculated at one standard deviation below and above the mean of social jetlag and chronotype. Lower and higher social jetlag correspond to 1.78 and 2.70 hours, respectively. Lower MEQ-5 scores indicate greater eveningness, whereas higher scores indicate greater morningness. Higher PSQI scores indicate poorer sleep quality. Estimates were adjusted for physical activity, bedtime procrastination, sex, age, caffeine intake frequency, smoking or e-cigarette use frequency, alcohol use frequency, academic stress, and dormitory environmental disturbance.

## 4 Discussion

This study examined the associations among physical activity, bedtime procrastination, social jetlag, chronotype, and sleep quality among college students. Higher physical activity was directly associated with lower PSQI scores and was indirectly associated with PSQI scores through bedtime procrastination. The indirect associations involving social jetlag varied by chronotype. They were negative at more evening-oriented and mean chronotype scores but reversed direction at a more morning-oriented chronotype score. Chronotype also moderated the association between social jetlag and PSQI scores. However, the pattern observed at greater morningness differed from the hypothesized direction. These findings describe a cross-sectional association structure and do not establish temporal or causal relationships.

### 4.1 Physical activity and sleep quality

Physical activity remained negatively associated with PSQI scores after adjustment for demographic, lifestyle, academic, and dormitory-related covariates. This result is consistent with previous evidence among university students (14). It also agrees with recent research linking higher physical activity to lower PSQI scores among Chinese college students (16). Together, these findings support a reliable association between physical activity and fewer self-reported sleep quality problems. This association may involve more than the energy expenditure of exercise. The two-process model proposes that sleep is regulated by both homeostatic sleep pressure and circadian timing (17). Physical activity may form part of a more structured daytime routine. Students who are more active may have clearer boundaries between activity and rest and more regular daily schedules. In contrast, lower activity may occur with prolonged sedentary time and later evening wakefulness. These patterns may help explain why physical activity was associated with lower PSQI scores. The relationship may also be bidirectional. Better sleep may provide students with more energy and greater motivation to participate in physical activity. Previous reviews have similarly noted that physical activity may be related to subsequent sleep, while sleep status may also influence later activity (9, 10). The present results therefore support an association between physical activity and sleep quality, but do not determine which occurs first.

### 4.2 Bedtime procrastination in the association between physical activity and sleep quality

Bedtime procrastination was involved in the indirect association between physical activity and PSQI scores. Higher physical activity was associated with less bedtime procrastination, and less bedtime procrastination was associated with lower PSQI scores. This result is consistent with previous studies linking bedtime procrastination to poorer sleep quality among college students (21). Similar findings have been reported in research on mobile phone dependence and sleep hygiene (22, 23). Bedtime procrastination reflects difficulty acting on the intention to go to sleep (18). College students often have considerable control over their evening schedules. Smartphones, gaming, videos, social activities, and unfinished academic work may delay sleep preparation. The problem is therefore not simply a preference for a late bedtime. It also concerns the ability to stop ongoing activities and begin preparing for sleep. The association between physical activity and bedtime procrastination may reflect several behavioral processes. Exercise is often arranged around classes, planned sessions, or regular routines, which may create clearer boundaries between daytime activity and evening rest. In addition, greater physical activity may increase physiological sleep pressure or perceived tiredness later in the day, which could make it easier to disengage from evening activities and go to bed as intended. Conversely, students with lower physical activity may have less structured routines and more prolonged sedentary time at night. These explanations remain tentative because the present cross-sectional study did not assess evening fatigue, sleep pressure, or the timing of physical activity. Bedtime procrastination may therefore represent one behavioral context in which physical activity and sleep quality are related.

### 4.3 Social jetlag in the association between physical activity and sleep quality

Social jetlag was involved in a conditional indirect association between physical activity and PSQI scores. Higher physical activity was associated with smaller social jetlag. At more evening-oriented and mean chronotype scores, smaller social jetlag was associated with lower PSQI scores. At a more morning-oriented chronotype score, however, the conditional association changed direction. Previous studies have linked social jetlag with depressive symptoms, sleep problems, and academic burnout among university students (30, 31). The present findings extend this evidence by placing physical activity within the same association model. University schedules may contribute to differences between school-day and free-day sleep timing. Classes often require early wake times, whereas free days allow students to sleep and wake later. Social activities, dormitory routines, and academic demands may further increase this difference. In the present study, social jetlag reflected the magnitude of the difference in sleep midpoint across the two types of days. The association between physical activity and social jetlag may reflect the regularity of daytime routines. Exercise can act as a non-photic cue for circadian timing, although its role depends on exercise timing and individual circadian state (28). Regular physical activity may also occur within more stable daily schedules. Such schedules may be associated with smaller changes in sleep timing across the week. Social jetlag may therefore represent one rhythm-related context in which physical activity and sleep quality are connected. However, the direction of this association differed across chronotype scores in the present model.

### 4.4 Bedtime procrastination and social jetlag as a serial indirect association

The serial indirect association through bedtime procrastination and social jetlag was statistically significant, but its direction varied by chronotype. Higher physical activity was associated with less bedtime procrastination, and less bedtime procrastination was associated with smaller social jetlag. At more evening-oriented and mean chronotype scores, this sequence was associated with lower PSQI scores. At a more morning-oriented chronotype score, the serial indirect estimate reversed direction. This pattern links bedtime behavior with the regularity of sleep timing across the week. Bedtime procrastination and social jetlag represent different but potentially connected processes. Bedtime procrastination concerns delayed sleep preparation on a given night. Social jetlag concerns differences in sleep timing between school days and free days. Repeated delays in bedtime may widen this difference, especially when students can wake later on free days but must wake early for classes. This serial pattern extends studies that have examined the two factors separately. It suggests that the association between physical activity and sleep quality may involve both bedtime self-regulation and weekly schedule regularity. However, direct evidence for the proposed order remains limited. The present result should therefore be viewed as an exploratory statistical sequence rather than a confirmed temporal chain. Longitudinal studies could test whether bedtime procrastination predicts later changes in social jetlag. Sleep diaries and objective sleep monitoring would also help clarify how nightly delays develop into differences between school-day and free-day sleep timing.

### 4.5 Chronotype as a moderator

Chronotype moderated the association between social jetlag and PSQI scores. Descriptively, neither types were the largest chronotype group in the present sample (40.5%), followed closely by evening types (38.7%), whereas morning types accounted for 20.8%. This distribution indicates a substantial eveningness tendency in the present sample. Because chronotype distributions vary across age groups and populations, and because the present study used convenience sampling, these proportions should be interpreted as sample-specific rather than as representative of the general population. At greater eveningness, larger social jetlag was associated with higher PSQI scores. The conditional indirect associations involving social jetlag showed a similar pattern. These findings are broadly consistent with the study hypothesis. One explanation concerns the fit between individual sleep preference and university schedules. Students with greater eveningness tend to prefer later sleep and wake times. Early classes and fixed morning schedules may require them to wake earlier than preferred on school days. They may then return to later schedules on free days. This pattern may make differences between school-day and free-day sleep timing more relevant to their sleep quality. Previous studies have also linked chronotype and social jetlag with sleep and psychological adjustment among university students (30, 31). The pattern at greater morningness was different. Under this condition, larger social jetlag was associated with lower PSQI scores. The conditional indirect estimates involving social jetlag also changed direction. This unexpected reversal should not be interpreted as evidence that greater social jetlag improves sleep quality among students with greater morningness. In the analytic data, free-day sleep midpoints were later than school-day sleep midpoints across participants. The reversal may instead reflect the restricted distribution of social jetlag, residual confounding, model-specific conditional estimation, or characteristics of the present sample. Unmeasured differences in class schedules, sleep duration, catch-up sleep, and weekend routines may also have contributed. The result should therefore be treated as an unexpected conditional association that requires replication. Future studies should use more diverse sleep-timing distributions and examine social jetlag together with sleep duration, class timing, and compensatory sleep. Chronotype was also weakly positively correlated with PSQI scores at the bivariate level. This result differs from studies that commonly associate eveningness with poorer sleep (45). However, the correlation was small and did not account for the other study variables. It also addressed a different question from the interaction analysis. The correlation described the overall association between chronotype and PSQI scores, whereas the interaction described how the association between social jetlag and PSQI scores varied by chronotype.

Overall, chronotype changed both the strength and direction of the associations involving social jetlag. The findings at greater eveningness supported the proposed risk pattern, but those at greater morningness did not. H4 and H5 were therefore partially supported. Future studies should distinguish positive and negative social jetlag and assess sleep duration, compensatory sleep, and class schedules. Larger samples and objective sleep measures are also needed to test whether this pattern is reproducible.

### 4.6 Implications for university sleep health

The findings suggest that university sleep health programs should consider more than sleep duration. Physical activity, bedtime procrastination, differences between school-day and free-day sleep timing, and chronotype may all help identify students who report sleep quality problems. These factors should be viewed as possible assessment indicators rather than confirmed intervention targets. Sleep health education may benefit from greater attention to bedtime behavior and weekly schedule regularity. Students could be encouraged to develop a consistent pre-sleep routine, limit stimulating activities before bedtime, and maintain relatively stable wake times across the week. Physical activity may also be considered within broader efforts to support regular daytime routines. Brief sleep diaries or self-assessment tools could help students recognize patterns of bedtime delay and large changes in sleep timing between school days and free days. Chronotype may also be relevant when providing sleep guidance. Students with greater eveningness may experience more difficulty aligning their preferred schedules with early classes and fixed morning demands. Guidance for these students could focus on gradual schedule adjustment, regular wake times, and the management of late-night activities. Chronotype itself should not be treated as a disorder. The aim should be to improve the fit between individual sleep preference and academic schedules where possible. These implications require further testing. The present study did not evaluate a sleep health program or determine whether changing these behaviors would improve sleep quality.

### 4.7 Limitations and future directions

Several limitations should be considered. First, the cross-sectional design does not establish temporal order or causality. The serial model was based on theory, but the proposed sequence cannot be confirmed from data collected at one time point. Longitudinal, cross-lagged, and intervention studies are needed to test how physical activity, bedtime procrastination, social jetlag, and sleep quality change over time. Second, the main variables were assessed through self-report. The results may therefore be affected by recall bias, response bias, and shared method variance. Harman's single-factor test does not rule out these problems. Future studies could combine questionnaires with accelerometers, sleep diaries, actigraphy, or other wearable measures. Third, social jetlag was calculated from self-reported sleep timing and may therefore contain estimation error. Its observed range was also relatively restricted in the present sample. Free-day sleep midpoints were later than school-day sleep midpoints across participants, meaning that the unexpected conditional pattern at more morning-oriented chronotype scores cannot be attributed simply to the use of an absolute difference. The restricted distribution, residual confounding, and sample-specific sleep schedules may have influenced the estimates. Future studies should assess social jetlag together with sleep duration, catch-up sleep, class timing, and objective sleep measures. Fourth, physical activity was represented by an overall PARS-3 score. The analysis did not distinguish activity type, timing, or specific intensity patterns. Morning, afternoon, and evening activity may have different associations with circadian timing and sleep. Objective monitoring could help clarify the role of activity timing and daily activity patterns. Fifth, convenience sampling was used in universities from four provinces in eastern China. The findings may not generalize to students in other regions or university settings. Some covariates were also measured with single items, and several possible confounders were not included. These include psychological distress, electronic media use, medication use, physical health, class schedules, and part-time work. In addition, the present conditional process model specified chronotype as a moderator of the social jetlag–PSQI association only. Possible moderation of the bedtime procrastination pathway by chronotype was not examined and warrants investigation in future studies. Future studies should use broader samples and more detailed measures of lifestyle, mental health, academic demands, and living conditions.

## 5 Conclusion

Higher physical activity was directly associated with lower PSQI scores among college students. It was also indirectly associated with lower PSQI scores through less bedtime procrastination. The indirect associations involving social jetlag depended on chronotype. At more evening-oriented and mean chronotype scores, higher physical activity was indirectly associated with lower PSQI scores through smaller social jetlag and through the serial pathway of less bedtime procrastination followed by smaller social jetlag. At a more morning-oriented chronotype score, both conditional indirect associations reversed direction. These reversed associations should be interpreted cautiously and require replication. Overall, the findings indicate that physical activity, bedtime procrastination, social jetlag, and chronotype are jointly related to sleep quality. Because the study was cross-sectional and relied mainly on self-report measures, the observed direct and indirect associations do not establish temporal or causal relationships.

## Data Availability

The raw data supporting the conclusions of this article will be made available by the authors, without undue reservation.
